# Sugar Profiling of Honeys for Authentication and Detection of Adulterants Using High-Performance Thin Layer Chromatography

**DOI:** 10.3390/molecules25225289

**Published:** 2020-11-13

**Authors:** Md Khairul Islam, Tomislav Sostaric, Lee Yong Lim, Katherine Hammer, Cornelia Locher

**Affiliations:** 1 Cooperative Research Centre for Honey Bee Products Limited (CRC HBP), University of Western Australia, Perth 6009, Australia; mdkhairul.islam@research.uwa.edu.au (M.K.I.); katherine.hammer@uwa.edu.au (K.H.); 2 Division of Pharmacy, School of Allied Health, University of Western Australia, Perth 6009, Australia; tom@chromatechscientific.com (T.S.); lee.lim@uwa.edu.au (L.Y.L.); 3 School of Biomedical Sciences, University of Western Australia, Perth 6009, Australia

**Keywords:** honey, sugar syrup, adulteration, Jarrah, Manuka

## Abstract

Honey adulteration, where a range of sugar syrups is used to increase bulk volume, is a common problem that has significant negative impacts on the honey industry, both economically and from a consumer confidence perspective. This paper investigates High-Performance Thin Layer Chromatography (HPTLC) for the authentication and detection of sugar adulterants in honey. The sugar composition of various Australian honeys (Manuka, Jarrah, Marri, Karri, Peppermint and White Gum) was first determined to illustrate the variance depending on the floral origin. Two of the honeys (Manuka and Jarrah) were then artificially adulterated with six different sugar syrups (rice, corn, golden, treacle, glucose and maple syrup). The findings demonstrate that HPTLC sugar profiles, in combination with organic extract profiles, can easily detect the sugar adulterants. As major sugars found in honey, the quantification of fructose and glucose, and their concentration ratio can be used to authenticate the honeys. Quantifications of sucrose and maltose can be used to identify the type of syrup adulterant, in particular when used in combination with HPTLC fingerprinting of the organic honey extracts.

## 1. Introduction

Honey has been regarded as nutritious food since ancient times [1,2], and it has also enjoyed increasing recognition for its bioactivities and potential medicinal applications. Monofloral honeys, in particular, have attracted good sale prices due to perceived higher bioactivity levels, which then led to these honeys being the subject of increasingly common adulterations [3,4,5]. The adulterations involve either the feeding of honeybees with sugar syrups or the deliberate addition of sugar syrups to the honey to increase the bulk weight [6,7,8,9]. Typical honey adulterants include sucrose syrup, high fructose corn syrup, maltose syrup, brown rice syrup, corn syrup, golden syrup, treacle syrup, glucose syrup, maple syrup, as well as industrial grade sugars like glucose and fructose. Syrups obtained from starch following enzymatic or acid treatment are also used [10,11,12,13].

The detection of these sugar-based adulterations is challenging, as honey is itself a highly concentrated sugar solution, with sugars accounting for about 80–85% of the total solids in most honeys. The main honey sugars are fructose and glucose, alongside much smaller amounts of disaccharides (e.g., sucrose, maltose, trehalose, turanose), trisaccharides (e.g., maltotriose, raffinose, erlose) and oligosaccharides [14]. Various methods are used for honey quality control and the detection of adulterants, such as C12/C13 isotope identification, fluorescence spectroscopy, high performance liquid chromatography (HPLC), ion exchange chromatography, gas chromatography, infrared spectroscopy, nuclear magnetic resonance spectroscopy and Raman spectroscopy [15,16,17,18,19,20]. However, these methods are not without challenges [21,22]. Sugars lack a chromophore and are poorly suited for methods that rely on detection by UV. Derivatisation into more easily detectable artefacts is possible; however, these approaches might be hampered by low sensitivity and poor selectivity [23]. Other methods like IR and NMR rely on the nonspecific detection of components and the establishment of adequate reference databases and threshold limits [24,25].

In this paper, we demonstrate the usefulness of a High-Performance Thin Layer Chromatography (HPTLC)-based method for the detection of sugar-based honey adulterants. It is a validated HPTLC method [26] that relies on the qualitative and quantitative analysis of glucose, fructose, maltose and sucrose in potentially adulterated honeys, complemented by HPTLC fingerprinting of the honey’s organic extract [27,28]. Taken together, the two levels of HPTLC analysis detect the natural product-based syrup adulterants (e.g., rice, maple, treacle, corn, golden syrup) and honeys that have been adulterated by adding pure glucose and fructose. The HPTLC analysis is cost-effective and easy to perform, yet also sophisticated as it can detect the presence of sugar-based honey adulteration, identifies the adulterant itself and estimates the level of adulteration.

## 2. Results and Discussion

### 2.1. Sugar Analysis of Honeys and Syrups

**Figure 1:**
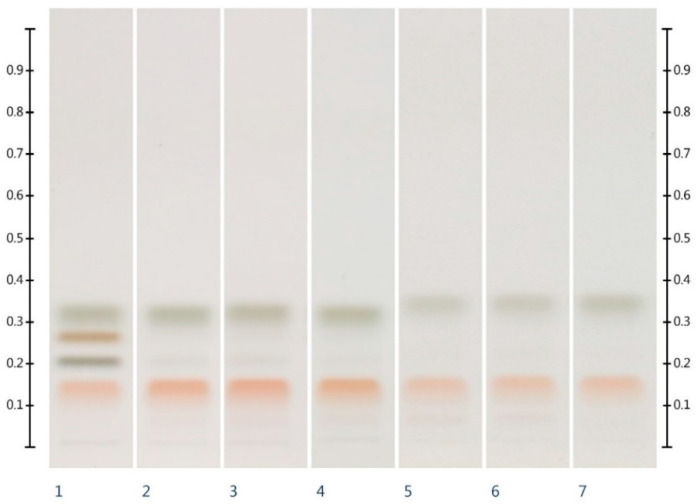
HPTLC images taken at T White light after derivatisation with aniline-diphenylamine-phosphoric acid reagent; Track 1—Standards (fructose, maltose, sucrose and glucose in increasing Rf values), Track 2—MAN, Track 3—JAR, Track 4—MAR, Track 5—KAR, Track 6—PEP, Track 7—WHG; Tracks 2–4 were obtained with 3 µL of aqueous methanolic honey solution, while Tracks 5–7 were obtained with 2 µL of aqueous methanolic honey solution.

Six honeys (Table 4) were analysed for their HPTLC sugar profile (Figure 1). Glucose presented as a green band with an Rf value of 0.32, fructose presented as an orange band with an Rf value of 0.14, sucrose presented as a dark brown band with an Rf value of 0.27, and maltose presented as a grey band with an Rf value of 0.20. Fructose and glucose contents were readily quantifiable in all honey samples (LOD/LOQ for glucose: 33.00/100.00 ng; LOD/LOQ for fructose: 21.98 ng/66.62 ng [26]), but maltose and sucrose, if present, were below the limits of detection and quantification (for sucrose 21.17 ng (LOD) and 64.15 ng (LOQ) and for maltose 63.51 ng (LOD) and 192.45 ng (LOQ) [26]). The fructose to glucose ratio (F:G), which is an important parameter for honey authentication and also a predictor of a honey’s tendency to crystallise [29,30,31], was calculated from these findings (Table 1).

**Figure 2:**
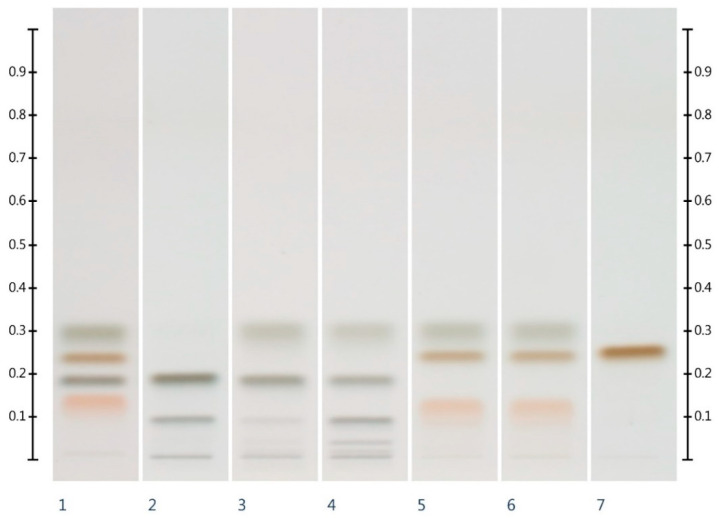
HPTLC images taken at T White light after derivatisation with aniline-diphenylamine-phosphoric acid reagent; Track 1—Standards (fructose, maltose, sucrose and glucose in increasing Rf values), Track 2—COR, Track 3—RIC, Track 4—GLU, Track 5—GOL, Track 6—TRE, Track 7—MAP; Tracks 2–7 were obtained with a 3 µL aqueous methanolic syrup solution.

Six syrups (Table 2) were also analysed for their HPTLC sugar profile (Figure 2) and fructose, glucose, sucrose and maltose contents. All syrups were found to contain one or more of these sugars within detectable limits (Table 3). As all the syrups contain a sugar other than glucose and fructose (i.e., sucrose or maltose), the adulteration of honey with these syrups should be detectable and the adulteration levels estimated.

### 2.2. Sugar Analysis of Adulterated Honeys

**Figure 3:**
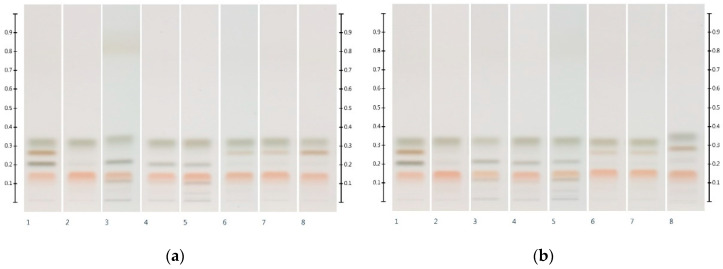
HPTLC images taken at T White light after derivatisation with aniline-diphenylamine-phosphoric acid reagent; (**a**) Track 1—Standards (fructose, maltose, sucrose and glucose in increasing Rf values), Track 2—MAN, Track 3—MAN-COR 30%, Track 4—MAN-RIC 30%, Track 5—MAN-GLU 30%, Track 6—MAN-GOL 30%, Track 7—MAN-TRE 30%, Track 8—MAN-MAP 30%; (**b**) Track 1—Standards (fructose, maltose, sucrose and glucose in increasing Rf values), Track 2—JAR, Track 3—JAR-COR 30%, Track 4—JAR-RIC 30%, Track 5—JAR-GLU 30%, Track 6—JAR-GOL 30%, Track 7—JAR-TRE 30%, Track 8—JAR-MAP 30%. Honey samples were analysed as a 3 µL aqueous methanolic honey solution.

Figure 3 shows the HPTLC sugar profiles of Manuka and Jarrah honeys artificially adulterated with 30% *w*/*w* of the respective six syrups. Honeys adulterated with corn, rice and glucose syrups presented with an additional maltose band at Rf 0.20 (as well as additional bands of unidentified compounds at Rf ≤ 0.11). Honeys adulterated with golden, treacle and maple syrups presented with an additional sucrose band at Rf 0.27. The level of adulteration, as well as the F:G ratio of the adulterated samples, was estimated by quantifying the respective sugar contents (Table 3).

A one-way ANOVA confirmed that there was a significant difference (*p* < 0.05) between the individual sugar contents of the respective honeys and the corresponding adulterated samples. A comparison of the F:G ratios showed a decrease in the ratio by about a third in the honeys adulterated with corn, rice and glucose syrups (Table 3) when compared with the respective unadulterated honey samples (Table 1). This correlated with the substitution of 30% *w*/*w* of the honeys with the respective syrups. Furthermore, the adulterated honeys showed detectable maltose levels, with the lowest in honeys adulterated with glucose syrup, followed by honeys adulterated with rice and corn syrups.

The highest F:G ratios were obtained for honeys adulterated with maple syrup, and these honeys also exhibited high levels of sucrose. The honeys adulterated with golden and treacle syrups showed F:G ratios closer in values to those of the respective unadulterated honeys; however, the sucrose levels in these adulterated honeys were elevated above the limit of detection.

**Figure 4:**
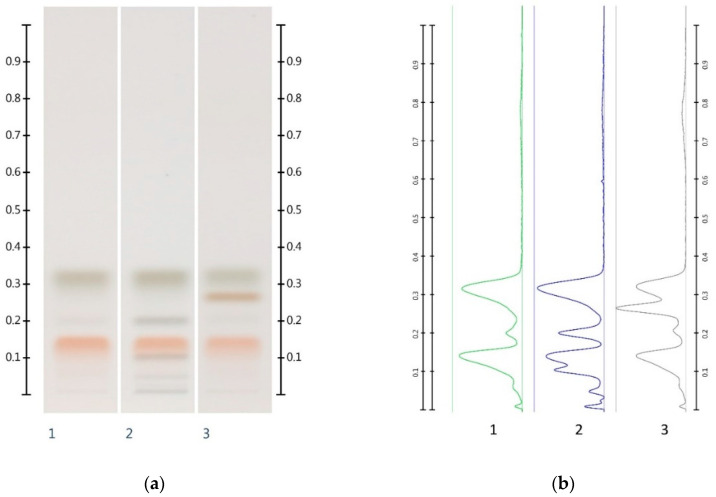
Images taken at T White light; (**a**) Track 1—MAN, Track 2—MAN-GLU 30%, Track 3—MAN-MAP 30%; (**b**) their respective chromatograms.

It needs to be noted, however, that some of the above values deviate from their respective theoretical values (as indicated in brackets in Table 3). A number of reasons for these discrepancies can be proposed. In the case of samples adulterated with corn, rice or glucose syrup, the presence of unidentified compounds might lead to a partial band overlap with the sugar band, leading to an overestimation of the respective sugar content. High levels of sucrose and maltose found in the adulterated syrups might cause a potential coelution with either glucose or fructose, causing inaccuracies in their quantification (Figure 4). Moreover, maltose and sucrose, which are naturally present in unadulterated honeys [14] but in quantities below the limits of detection and quantification, could have added to the peak areas and contributed to a higher than estimated amount of maltose and/or sucrose in the adulterated honey samples.

Notwithstanding these limitations, in a commercial context, the identification of an adulteration would be more important and also more relevant than the exact quantification of the level of adulteration. Thus, the proposed method, even though it provides only an estimate and not an accurate quantification of the level of adulteration, is still of significant value to the honey industry.

### 2.3. Organic Extract Analysis of Honeys, Syrups and Adulterated Honeys

On the basis of the HPTLC sugar analysis alone, it would be challenging to differentiate between honey adulterations using either golden or treacle syrups, given the similarities in their sugar profiles. To provide a more in-depth investigation of the type of adulterant, we chose to add another level of analysis by focusing on the honeys’ nonsugar HPTLC fingerprints. Previous studies have demonstrated that the HPTLC fingerprint of a honey’s dichloromethane extract provides a unique signature that can assist in the authentication of a honey’s floral source [27,28]. When the same analytical approach is applied to sugar syrups as well as the adulterated honey samples, the resulting fingerprints at 254 nm, at 366 nm prior to and after derivatisation with vanillin reagent, as well as at white light after derivatisation were found to also assist with the detection of honey adulterations.

The HPTLC analysis of the organic extract of Manuka honey (MAN) was characterised by two major bands (Rf 0.35 and Rf 0.52) at 254 nm, three major bands (Rf 0.15, Rf 0.25 and Rf 0.33) at 366 nm, four major bands (Rf 0.23, Rf 0.34, Rf 0.41 and Rf 0.46) at white light after derivatisation, as well as four major bands (Rf 0.15, Rf 0.22, Rf 0.33 and Rf 0.44) at 366 nm after derivatisation.

The Jarrah honey (JAR) extract presented three major bands (Rf 0.30, Rf 0.40 and Rf 0.44) at 254 nm, two major bands (Rf 0.09 and Rf 0.31) at 366 nm, four major bands (Rf 0.23, Rf 0.37, Rf 0.40 and Rf 0.48) at white light after derivatisation, as well as four major bands (Rf 0.30, Rf 0.36, Rf 0.39 and Rf 0.46) at 366 nm after derivatisation.

**Figure 5:**
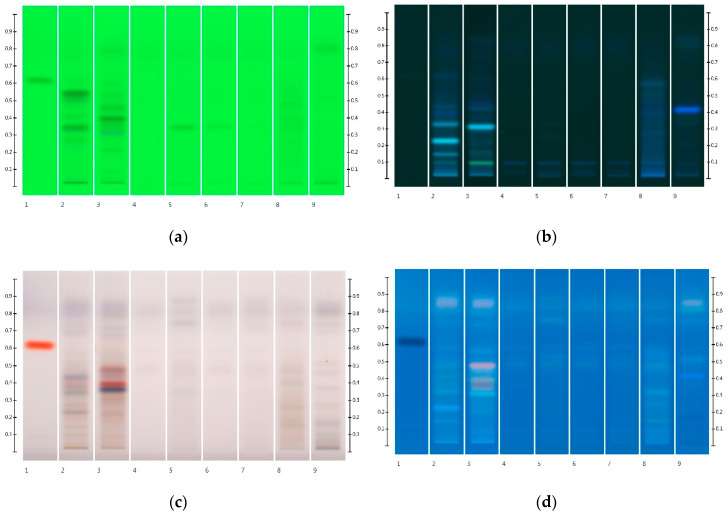
Images taken at (**a**) 254 nm; (**b**) 366 nm; (**c**) White light after derivatisation and (**d**) 366 nm after derivatisation with vanillin reagent; Track 1—4,5,7-trihydroxyflavanon, Track 2—MAN, Track 3—JAR, Track 4—COR, Track 5—RIC, Track 6—GLU, Track 7—GOL, Track 8—TRE, Track 9—MAP; 5 µL honey and syrup extracts respectively.

**Figure 6:**
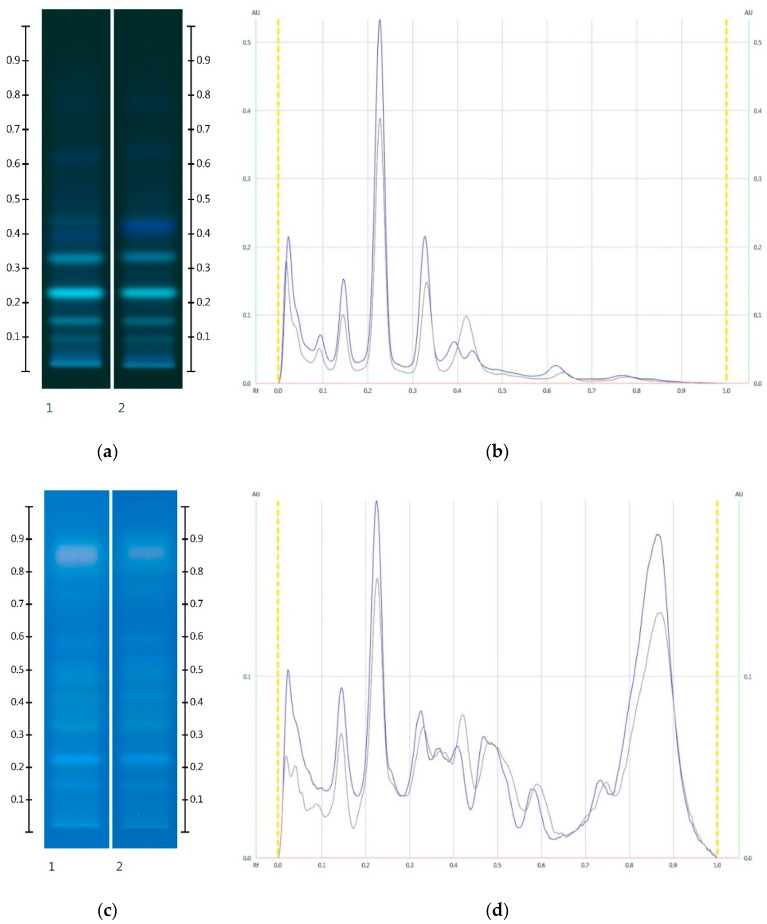
Images taken at (**a**) 366 nm after development and (**b**) the respective chromatograms; (**c**) 366 nm after derivatisation with vanillin reagent and (**d**) the respective chromatograms; Track 1—MAN, and Track 2—MAN-MAP 30%; 5 µL extract respectively. (Green colour—MAN, and Black colour—MAN-MAP 30%).

Conversely, corn, golden and treacle syrup extracts did not present any significant HPTLC bands at any wavelength, although the latter exhibited a strong fuzzy background, in particular after derivatisation. Rice and glucose syrup extracts produced a single faint band (Rf 0.32) at 254 nm, whereas maple syrup extract showed a strong fluorescent band (Rf 0.41) at 366 nm (Figure 5). This characteristic band was also seen in honeys adulterated with maple syrup (MAN-MAP 30% and JAR-MAP 30%, Figure 6) at Rf 0.41 at 366 nm.

**Figure 7:**
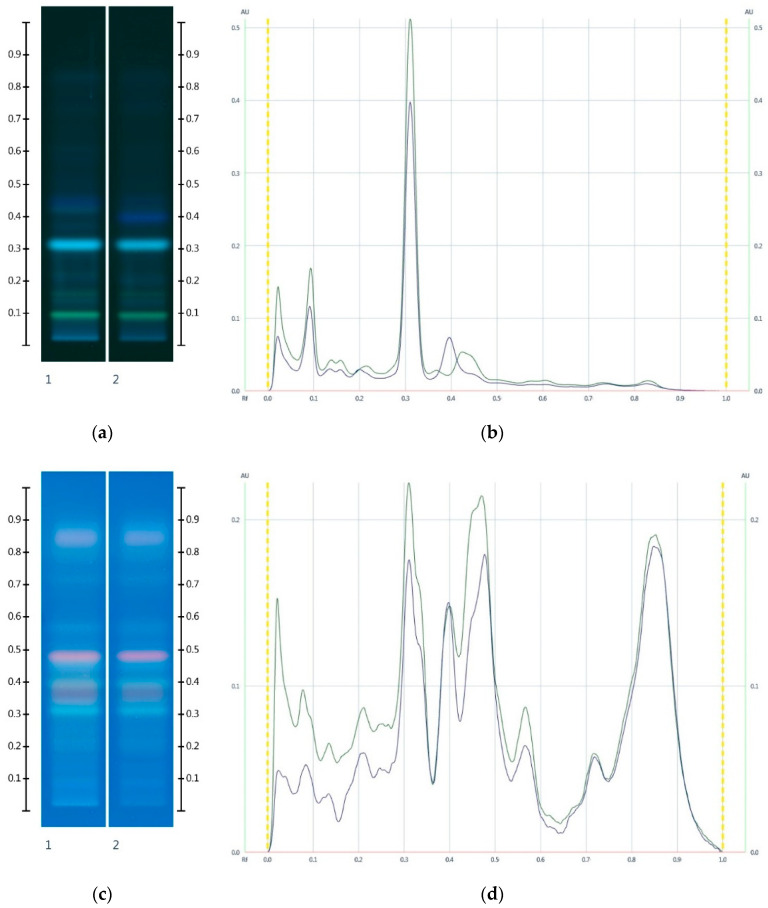
Images taken at (**a**) 366 nm and (**b**) the respective chromatograms; (**c**) 366 nm after derivatisation with vanillin reagent and (**d**) the respective chromatograms; Track 1—JAR, and Track 2—JAR-MAP 30%; 5 µL extract respectively. (Green colour—JAR, and Black colour—JAR-MAP 30%).

Unlike maple syrup, corn, rice, glucose, golden and treacle syrup extracts did not have any major bands that would allow their detection in adulterated honey samples. Nonetheless, adulterations with these syrups resulted in the HPTLC fingerprint appearing ‘paler’ with less strongly developed band intensities compared to the respective unadulterated honey (Figure 6 and Figure 7). The reason for the ‘paler’ fingerprint profile is the extraction protocol, which results in a less concentrated organic extract for the adulterated honey samples.

**Figure 8:**
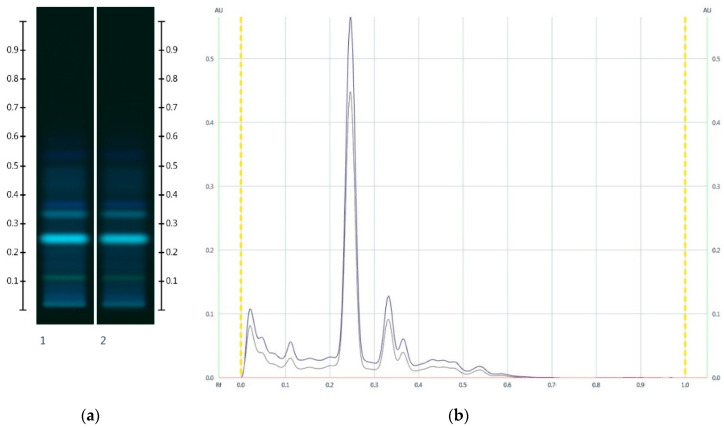
Images taken at (**a**) 366 nm and (**b**) the respective chromatograms; Track 1—JAR, Track 2—JAR-Artificial JAR 30%; 5 µL extract respectively. (Green colour—JAR, and Black colour—JAR-Artificial JAR 30%).

On this basis, even honey adulterations with pure fructose and glucose (in an attempt to maintain the same F:G ratio as that found in the unadulterated honey) can be detected. While the HPTLC sugar analysis of such a sample would be unable to identify the adulteration, the corresponding honey extract fingerprint will appear ‘paler’ due to reduced band intensities and its chromatogram will display reduced peak intensities (Figure 8), and this in itself would raise concern about the possibility of an adulteration.

The HPTLC fingerprint of the organic extract and the corresponding chromatogram of Manuka honeys (Figure 6) showed a noticeable drop in peak intensity in the adulterated MAN-MAP 30% at Rf 0.15, 0.23 and 0.33 at 366 nm prior to and after derivatisation when compared to MAN. Additionally, a new band appeared at Rf 0.41 at 366 nm both prior to and after derivatisation in the adulterated sample. Maple syrup also presented this major band.

The HPTLC fingerprint of the organic extract and the corresponding chromatogram (Figure 7) also demonstrated a noticeable drop in peak intensity in the adulterated Jarrah honey sample: The peak intensities of JAR-MAP 30% at Rf 0.09 and 0.31 at 366 nm and at Rf 0.31, 0.47 and 0.56 at 366 nm after derivatisation decreased noticeably when compared to JAR. An additional band at Rf 0.41 at 366 nm, characteristic of Maple syrup, was also evident.

The collective data suggests that a reduction in peak intensity in the respective organic extract chromatogram can also be used to detect adulterations of honeys with glucose and fructose in the same ratio as that naturally present in honey. This is further illustrated with a JAR honey sample artificially adulterated with 30% *w*/*w* of a fructose-glucose 1:3 mixture, for which a noticeable reduction in the major peak intensity is also seen (Figure 8).

In summary, the respective HPTLC sugar profile and the corresponding quantification of glucose, fructose, maltose and sucrose serve as one level of analysis, whereas the careful inspection of the HPTLC fingerprint of the honey sample’s organic extract profile offers an additional dimension. It has previously been shown that the HPTLC analysis of the organic honey extract can be used to authenticate a honey’s floral sources [27,32]. In this study, we demonstrate that the extract can also be used to detect the presence of sugar syrup adulterants when used in combination with HPTLC sugar profiling.

## 3. Materials and Methods

### 3.1. Chemicals and Reagents

The chemicals and reagents were sourced from: Glucose, sucrose, 1-butanol (Chem-Supply Pty Ltd., Gillman, Australia), fructose, aniline (Sigma-Aldrich, St. Louis, MO, USA), boric acid (Pharma Scope, Welshpool, Australia), Methanol (Scharlau, Barcelona, Spain), 2-propanol (Asia Pacific Specialty Chemicals Ltd., Sydney, Australia), diphenylamine, phosphoric acid (Ajax Finechem Pvt Ltd., Sydney, Australia) and 4,5,7-trihydroxyflavanone (Alfa Aesar, England, UK). Commercial syrups and honeys (Table 4) were obtained from beekeepers and supermarkets in Western Australia.

### 3.2. Standards and Reagent Preparation

To prepare the glucose, fructose, sucrose and maltose standard solutions, 25 mg of the respective sugars were dissolved in 100 mL of 50% aqueous methanol. To prepare the derivatisation reagent, 2 g of diphenylamine and 2 mL of aniline were dissolved in 80 mL of methanol, 10 mL of phosphoric acid (85%) were added and the solution was made up to 100 mL using methanol. A solution of 0.5 mg/mL of 4,5,7-trihydroxyflavanone in methanol was prepared as a reference solution. The vanillin derivatisation reagent was prepared by dissolving 0.5 g of vanillin in 50 mL of ethanol, followed by the dropwise addition of 1 mL of sulfuric acid.

### 3.3. Sample Preparation

Adulterated honey samples were prepared by mixing the respective syrup (rice, corn, golden, treacle, glucose and maple) with honey (Manuka and Jarrah) to a concentration of 30% (*w*/*w*). The samples were heated for about 30 min at 36 °C in a water bath to assist with the mixing into homogenous blends.

For the analysis, 100 mg of each sample (six honeys, six syrups and 12 adulterated honeys) were dissolved in 100 mL of 50% aqueous methanol.

To prepare the organic honey extracts, approximately 1 g of each sample (six honeys, six syrups and 12 adulterated honeys) was mixed with 2 mL of deionized water and vortexed to produce a homogenous solution. The aqueous solution was then extracted three times with 5 mL of dichloromethane. The combined organic extracts were dried with anhydrous MgSO_4_, filtered, and the solvent was evaporated at ambient temperature. The extracts were stored at 4 °C until further analysis.

### 3.4. Instrumentation and High-Performance Thin Layer Chromatography (HPTLC) Method

#### 3.4.1. Sugar Analysis

All standards and samples were applied as 8-mm bands 8 mm from the lower edge of the HPTLC plate using a semiautomated HPTLC application device (Linomat 5, CAMAG, Muttenz, Switzerland) set at a speed of 50 nLs^−1^. The glucose, fructose, sucrose and maltose standard curves were obtained by applying 1 µL, 2 µL, 3 µL, 4 µL and 5 µL of the respective standard solutions (calibration curves available in the Supplementary Material section). For the analysis of sugars in the honey, syrup and adulterated honey samples, 3 µL of each sample solution were applied.

The chromatographic separation was performed at ambient temperature on silica gel 60 F_254_ HPTLC plates (glass plates 20 × 10 cm) in a saturated (33% relative humidity) automated development chamber (ADC2, CAMAG). The development chamber was saturated for 60 min, and the HPTLC plates were presaturated with mobile phase (1-butanol: 2-propanol: aqueous boric acid (5 mg/mL) 30:50:10 *v*/*v*/*v*) for 5 min. The plates were automatically developed to a distance of 85 mm, and after drying for 5 min they were analyzed under white light using an HPTLC imaging device (TLC Visualizer 2, CAMAG). The chromatographic images were digitally processed using specialized HPTLC software (visionCATS, CAMAG) [27,28,32].

After documentation of the initial chromatographic results, the HPTLC plates were derivatised with 2 mL of the aniline-diphenylamine-phosphoric acid reagent (CAMAG Derivatiser). After heating for 10 min at 115 °C (CAMAG TLC Plate Heater III) and cooling to room temperature, the plates were re-analysed under transmission white (T white) light using the HPTLC imaging device.

#### 3.4.2. Organic Extract Analysis

The obtained organic extracts were reconstituted in 100 μL of dichloromethane prior to the HPTLC analysis.

The reference standard (4 µL) and the organic extracts (5 µL) were applied as 8-mm bands at 8 mm from the lower edge of the HPTLC plate at a rate of 150 nLs^−1^ using a semiautomated HPTLC application device (Linomat 5, CAMAG).

The chromatographic separation was performed at ambient temperature on silica gel 60 F_254_ HPTLC plates (glass plates 20 × 10 cm) in a saturated (33% relative humidity) automated development chamber (ADC2, CAMAG) using toluene: ethyl acetate: formic acid (6:5:1 *v*/*v*/*v*) as mobile phase. The instrument was used at the default set-up mode and developed to a distance of 70 mm at room temperature. The obtained chromatographic results were documented under white light, 254 nm and 366 nm respectively, using an HPTLC imaging device (TLC Visualizer 2, CAMAG).

After initial documentation of the chromatographic results, each plate was derivatised with 3 mL of vanillin reagent. The derivatised plates were heated for 3 min at 115 °C (CAMAG TLC Plate Heater III) and then cooled to room temperature and analysed under white light and 366 nm with the HPTLC imaging device.

### 3.5. Statistical Analysis

All experiments were performed in triplicates, and the obtained quantitative results were expressed as the mean values of three determinations. The differences in the data generated for pure and adulterated honeys were then evaluated by a one-way analysis of variance (ANOVA), where a *p*-value of less than 0.05 was considered statistically significant.

## 4. Conclusions

In this paper, two HPTLC-based analysis methods were investigated. In combination, they allow the determination of honey’s major sugar profile and detect sugar syrup-based adulterations. For common adulterants like glucose, fructose, brown rice, corn starch, maltose, treacle or maple syrups, which contain only one, two or a combination of common sugars, HPTLC sugar profiling alone may be sufficient to detect the adulteration due to the presence of noticeable quantities of sucrose and/or maltose. Our analysis further demonstrates that even if the adulterants consist of only glucose and fructose that are added to imitate the fructose to glucose ratios of naturally occurring honeys, their deliberate addition to the honey can be detected via reduced peak intensities in the HPTLC profile of the organic extract.

In conclusion, the quantification of glucose, fructose, sucrose and maltose via a validated HPTLC analysis complemented by the HPTLC fingerprinting of an organic sample extract has allowed for the detection of adulteration, the identification of syrup adulterants, as well as an estimation of the level of adulteration. The developed HPTLC method is simple, easy to perform and cost-effective, and as such it can assist the honey industry in its quality control. Moreover, while only honey samples were investigated in this paper, it can be assumed that the proposed method has applications that can be extended beyond honey to other food and natural products that contain sugars.

## Figures and Tables

**Table 1:** Fructose and glucose content, and fructose to glucose ratio (F:G) of honeys ^1^.

Honey	ID	Fructose (mg/g)	Glucose (mg/g)	F:G Ratio
Manuka	MAN	392	307	1.3
Jarrah	JAR	426	337	1.3
Marri	MAR	439	269	1.6
Karri	KAR	387	249	1.5
Peppermint	PEP	404	257	1.5
White Gum	WHG	415	323	1.3

^1^ Values represent the mean of triplicate data. One-way ANOVA confirmed no significant difference between replicates (*p* > 0.05).

**Table 2:** Glucose, fructose, sucrose and maltose content of syrups ^1^.

Syrup	ID	Glucose (mg/g)	Fructose (mg/g)	Sucrose (mg/g)	Maltose (mg/g)	Remarks
Corn	COR	-	-	-	372	Contains additional unidentified compounds
Rice	RIC	250	-	-	242	Contains additional unidentified compounds
Glucose	GLU	178	-	-	148	Contains additional unidentified compounds
Golden	GOL	200	189	206	-	-
Treacle	TRE	191	185	204	-	-
Maple	MAP	-	-	486	-	-

^1^ Values represent the mean of triplicate data. One-way ANOVA confirmed no significant difference between replicates (*p* > 0.05).

**Table 3:** Experimentally determined content ^1^ (and variation from the theoretical value) of various sugars in artificially adulterated Manuka (MAN) and Jarrah (JAR) honeys, and their fructose to glucose ratio (F:G).

Samples	Glucose (mg/g)	Fructose (mg/g)	Sucrose (mg/g)	Maltose (mg/g)	F:G Ratio
MAN Corn 30%	196 (−9%)	185 (−33%)	-	124 (+11%)	0.9
MAN Rice 30%	281 (−3%)	216 (−21%)	-	90 (+24%)	0.8
MAN Glucose 30%	348 (+ 30%)	262 (−4%)	-	79 (+79%)	0.8
MAN Golden 30%	243 (−12%)	332 (0%)	68 (+11%)	-	1.4
MAN Treacle 30%	262 (−4%)	332 (+1%)	67 (+10%)	-	1.3
MAN Maple 30%	176 (−18%)	269 (−2%)	166 (+14%)	-	1.5
JAR Corn 30%	204 (−14%)	210 (−29%)	-	123 (+10%)	1.0
JAR Rice 30%	302 (−3%)	234 (−21%)	-	93 (+29%)	0.8
JAR Glucose 30%	257 (−11%)	206 (−31%)	-	58 (+31%)	0.8
JAR Golden 30%	242 (−18%)	358 (+1%)	72 (+17%)	-	1.5
JAR Treacle 30%	234 (−20%)	333 (−6%)	77 (+25%)	-	1.4
JAR Maple 30%	215 (−9%)	338 (+13%)	153 (+5%)	-	1.6

^1^ Values represent the mean of triplicate data. One-way ANOVA confirmed no significant difference between replicates (*p* > 0.05).

**Table 4:** Honey samples and commercial syrups.

	Type	Label and Packaging Information	Sample ID
Honey	Manuka	Australian Manuka (MGO 514+) Barnes Naturals Pty Ltd.	MAN
	Jarrah	Boyanup Jarrah Sweet As Apiary, WA	JAR
	Marri	Pooled Sample ^1^ (*n* = 12)	MAR
	Karri	Pooled Sample ^1^ (*n* = 9)	KAR
	Peppermint	Pooled Sample ^1^ (*n* = 4)	PEP
	White Gum	Pooled Sample ^1^ (*n* = 7)	WHG
Syrups	Rice	Organic Rice Syrup Pureharvest, VIC	RIC
	Corn	Corn Malt Syrup Korea Connections Ptl Ltd.	COR
	Golden	CSR Golden Syrup Sugar Australia Pty Ltd.	GOL
	Treacle	CSR Treacle Syrup Sugar Australia Pty Ltd.	TRE
	Glucose	Queen Glucose Syrup Queen Fine Foods Pty Ltd., QLD	GLU
	Maple	Queen Maple Syrup Dr. Oetker Queen Australia, QLD	MAP

^1^ Honey samples were collected from a range of beekeepers in Western Australia and blended in equal amounts to generate pooled samples in order to better reflect the typical phytochemical composition of these honeys.

## Supplementary Materials

The following are available online, Figure S1: Calibration Curves of Fructose (AU vs. Quantity), Figure S2: Calibration Curves of Glucose (AU vs. Quantity), Figure S3: Calibration Curves of Sucrose (AU vs. Quantity), Figure S4: Calibration Curves of Maltose (AU vs. Quantity).
