# Supplementary material for: Sugar Profiling of Honeys for Authentication and Detection of Adulterants Using High-Performance Thin Layer Chromatography
Source: Molecules. 2020 Nov 13;25(22):5289. doi: 10.3390/molecules25225289 (PMC7697932; doi:10.3390/molecules25225289)
Supplement: Supplementary file 1 [file molecules-25-05289-s001.pdf]

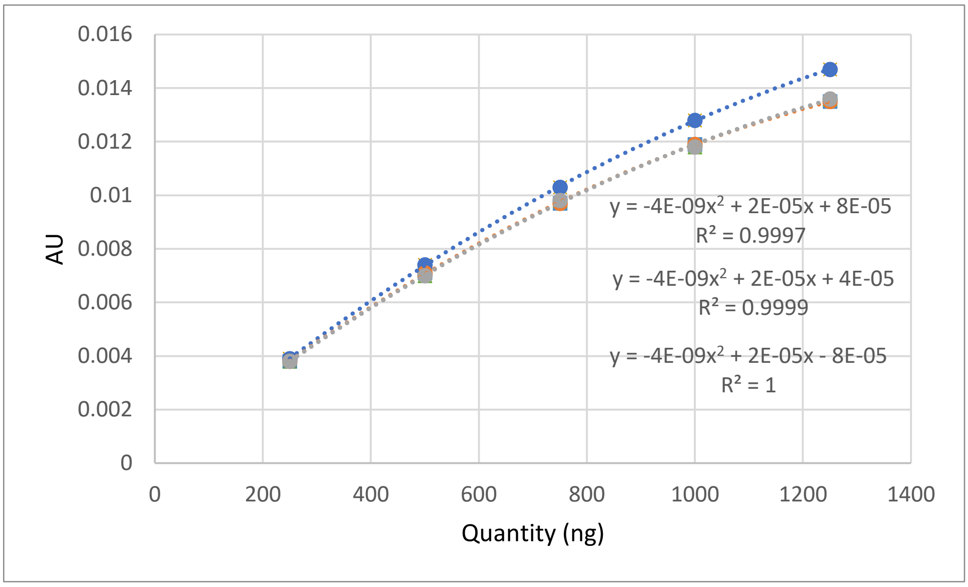

Figure S1. Calibration Curves of Fructose (AU vs Quantity).

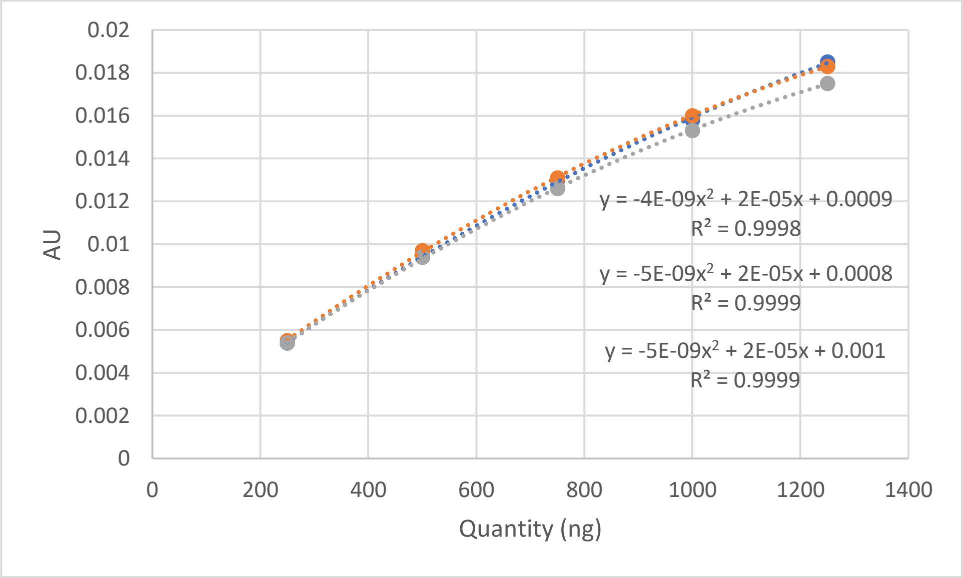

Figure S2. Calibration Curves of Glucose (AU vs Quantity).

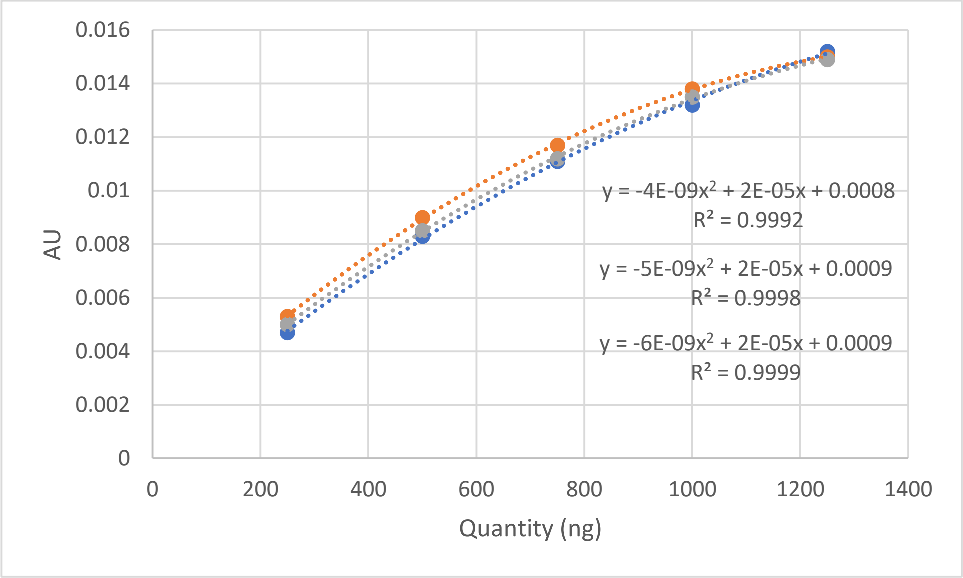

Figure S3. Calibration Curves of Sucrose (AU vs Quantity).

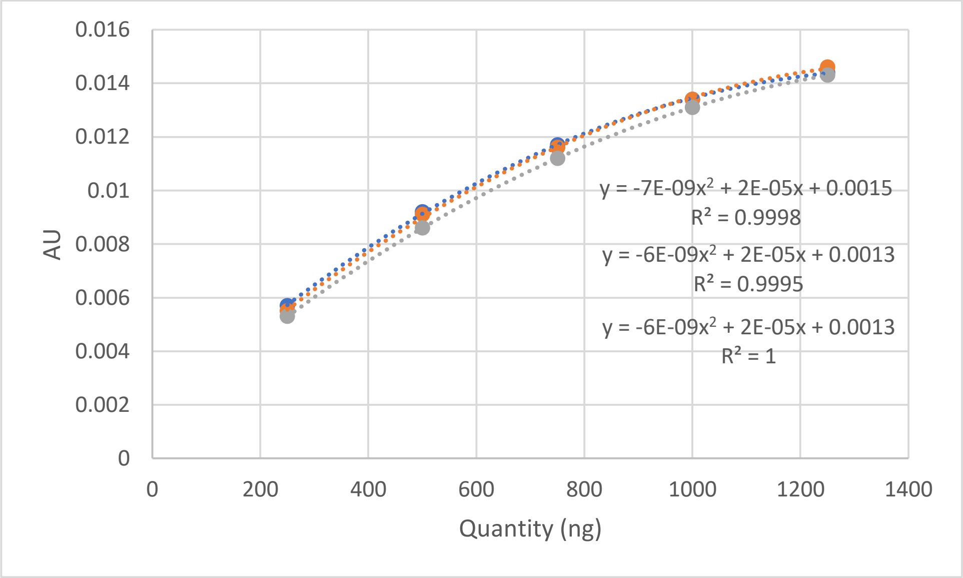

Figure S4. Calibration Curves of Maltose (AU vs Quantity).
